# Supplementary material for: A Highly Efficient Polystyrene-Based Cationic Resin to Reduce Bacterial Contaminations in Water
Source: Polymers (Basel). 2022 Nov 3;14(21):4690. doi: 10.3390/polym14214690 (PMC9654381; doi:10.3390/polym14214690)
Supplement: Supplementary file 1 [file polymers-14-04690-s001.zip › polymers-2008133-SM.pdf]

Supplementary Materials

A Highly Efficient Polystyrene-Based Cationic Resin to Reduce Bacterial Contaminations in Water

Anna Maria Schito [1,*], Debora Caviglia [1], Gabriella Piatti [1], and Silvana Alfei [2,*]

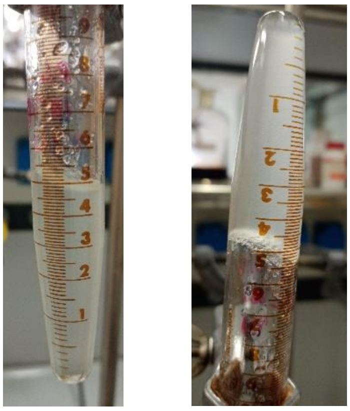

(**a**) (**b**)

Figure S1. Appearance of the swelled R4 obtained hydrating the dry resin with an excess of 10 mL of water and recovered by centrifugation at 4000 rpm for 30 min: test tube in normal vertical position (**a**) and in inverted position (**b**).

Table S1. Weights and cumulative weight loss (%) of R4 determined at times T_0-T_7.

	Time (h)	R4 (mg)	Weight loss (%)
T_0	0	267.3	0
T_1	1	154.8	42.1
T_2	1.5	92.0	65.6
T_3	2	50.4	81.1
T_4	2.5	22.8	91.4
T_5	3	8.9	96.7
T_6	3.5	8.4	96.8
T_7	6.5	9.0	96.6

Table S2. Weights of R4 at times T_0-T_3 and the cumulative swelling ratio percentages.

Time (min)		R4 (mg)	Swelling ratio (%)	Average equilibrium swellin ratio (%)
T_0	0	10.1	0	
T_1	13	373.2	3595.0	3399.3±158.9
T_2	30	333.9	3205.9	
T_3	45	353.2	3397.0	

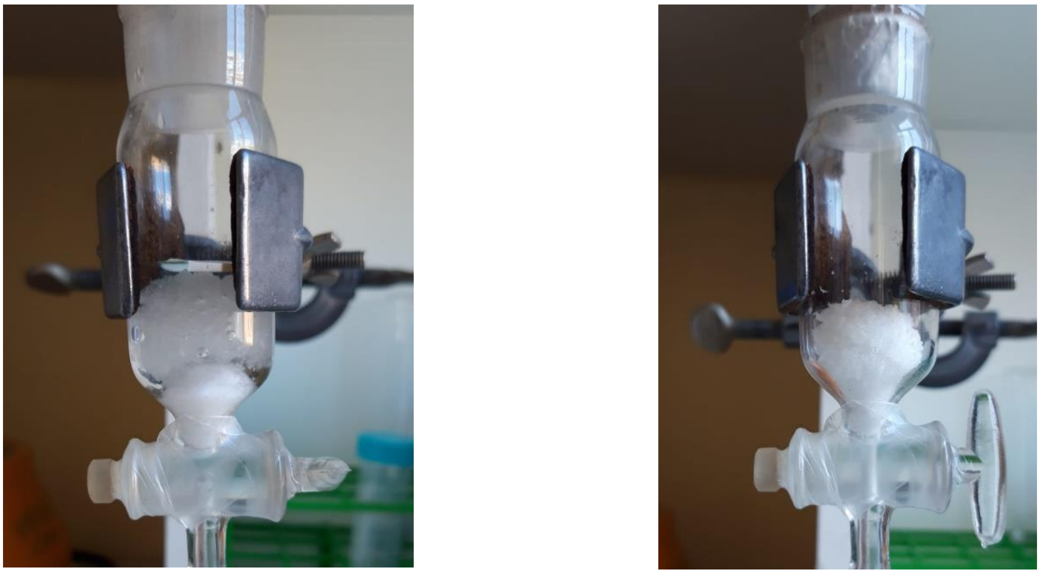

(**a**) (**b**)

Figure S2. Column filled with R4 when in contact with the aqueous model of contaminated water (**a**) and after filtration (**b**).

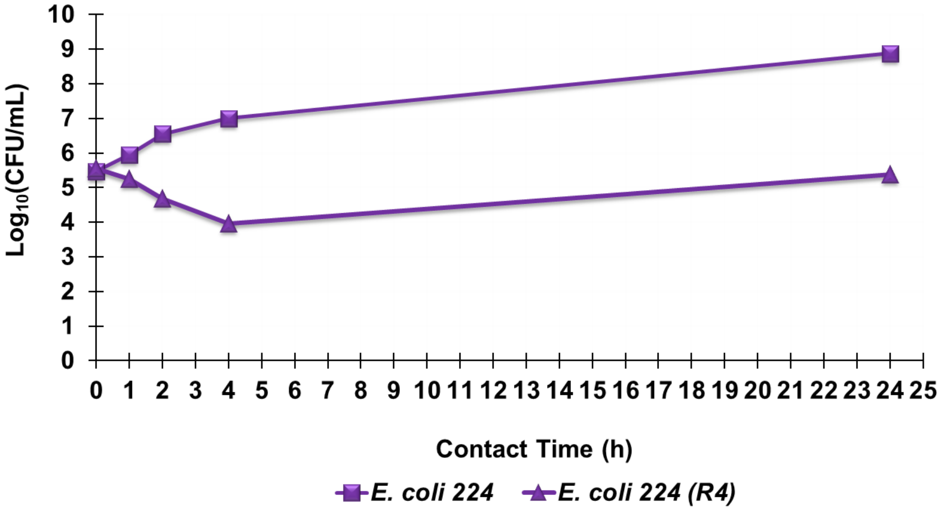

Figure S3. Variation in the Log_{10}(CFU/mL) of *E. coli* 224 when exposed to R4 [*E. coli* 224 (R4)] in a column imitating a sanitation system for 1, 2, 4 and 24 hours of exposure, vs. control (*E. coli* 224).

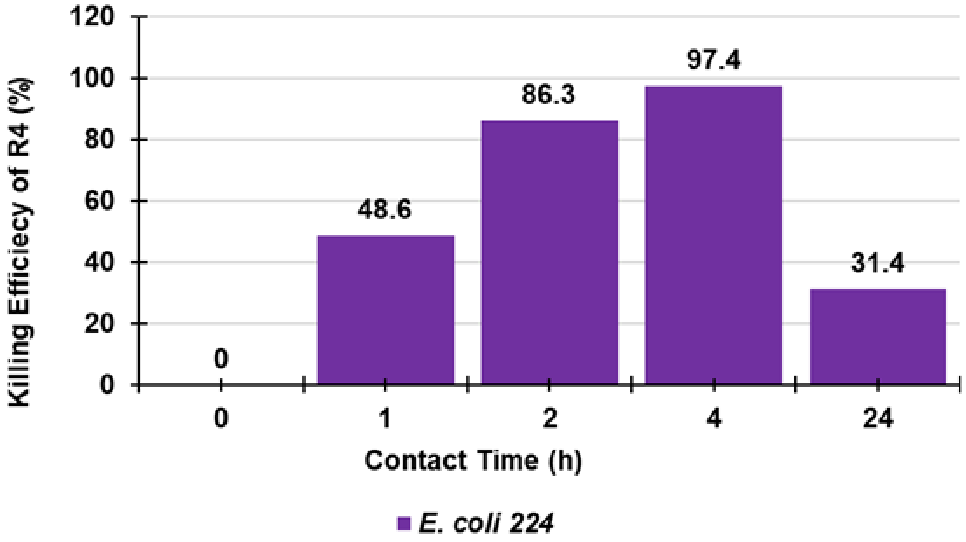

Figure S4. Percentage of the bacterial cells killed by R4 in a column imitating a sanitation system after 1, 2, 4 and 24 hours of exposure, when tested against *E. coli* 224.
